# Supplementary material for: Antibiotic (Mis)Use in COVID-19 Patients before and after Admission to a Tertiary Hospital in Serbia
Source: Antibiotics (Basel). 2022 Jun 24;11(7):847. doi: 10.3390/antibiotics11070847 (PMC9311622; doi:10.3390/antibiotics11070847)
Supplement: Supplementary file 1 [file antibiotics-11-00847-s001.zip › antibiotics-1771347-supplementary.pdf]

antibiotics MDPI

Supplementary File

Table S1. Serbian National guidelines for COVID-19 Treatment, Version 11. (Translated from Serbian to English and the original Serbian Version).

Protocol for the treatment of COVID-19 patients

Version 11

Form of the disease	Institution	Treatment
FORM 1<br>1. **Positive nasopharyngeal swab** (PCR for SARS-CoV-2, Ag test)<br>2. **Asymptomatic**<br>3. **Very mild clinical presentation**<br>4. **Patients without comorbidities** and with the mild form of the infection (hospitalized patients with sO_2>94% and **without signs of pneumonia on X-ray**)	**At-home treatment and home isolation** with monitoring and follow-up by **Covid clinic staff**	Vitamin therapy: alphacalcidol tablets (**Alpha D3**), 1x2 mcg, **vitamin C** 1x 1g (if there are no kidney issues)<br>**Do not routinely prescribe antibiotics due to the risk of serious adverse effects and the rising resistance to antimicrobial drugs*<br>**Antiviral therapy (to be introduced no later than the 5th day** of symptom onset): **favipiravir** tablets, 1,600 mg every 12 h on the first day, then 600 mg every 12 h for another 4 days (the drug is applied in patients with symptoms)<br>**Symptomatic treatment**
FORM 2<br>**1. Positive nasopharyngeal swab** (PCR for SARS-CoV-2, Ag test)<br>**2. Mild clinical presentation**<br>**3. Patients without comorbidities** and with the mild form of the infection (hospitalized patients with sO_2>94% and **with signs of pneumonia on X-ray, but without signs of hypoxia at admission**)	1. **Covid centers**: general wards<br>2. **General hospitals**: isolation units<br>Increased monitoring measures<br>Checking patient temperature, sO_2 and respiration rate several times a day	**Anticoagulant therapy:** low-molecular-weight heparin<br>**Antiviral therapy (to be introduced no later than the 5th day** of symptom onset): **favipiravir** tablets, 1,600 mg every 12 h on the first day, then 600 mg every 12 h for another 4 days<br>**Corticosteroid therapy**<br>**Vitamin therapy**: alphacalcidol tablets (**Alpha D3**), 1x2 mcg, **vitamin C** 1x1 g<br>**Convalescent plasma:** (within 2 weeks of symptom onset and after consultation with an infectologist and transfusiologist, according to the score)<br>*Application of antibiotics only in likely or proven bacterial infection*
FORM 3<br>**1. Positive nasopharyngeal swab** (PCR for SARS-CoV-2, Ag test)<br>**2. Moderate clinical presentation**<br>**3. Severe hypoxia** with necessary oxygen therapy (sO_2<90%), fever, multiple opacities on chest X-ray (or characteristic changes in the lungs visible on CT)<br>**4. Cytokine storm** (deterioration of general health status with ↑CRP,	1. **Covid centers**: semi-intensive care units<br>2. **General hospitals**: isolation units with constant monitoring (≤1h) of intensive care specialist, anesthesiologist, infectologist or internal medicine specialist<br>3. **In case of further deterioration transportation to Covid intensive care**	**Anticoagulant therapy:** low-molecular-weight heparin<br>**Vitamin therapy**: alphacalcidol tablets (**Alpha D3**), 1x2 mcg, **vitamin C** 1x1 g<br>**Oxygen therapy**: nasal catheter or mask O_2 (10 – 15 l/minute<br>**Antiviral therapy (if less than 5 days** have elapsed since symptom onset): **favipiravir** (as above) or **remdesivir** ampules 200 mg intravenously on the first day, then 100 mg IV/day for another 4 days

↑fibrinogen, ↑D-dimer, ↑IL-6) (at least one parameter elevated)		(**Corticosteroid therapy** and/or **tocilizumab** 8 mg/kg IV per dose. Two doses are administered (max 800 mg per dose)<br>**Antibiotic therapy**: in keeping with the guidelines for rational application of antibiotics<br>**Convalescent plasma:** (within 2 weeks of symptom onset and after consultation with an infectologist and transfusiologist, according to the score)
FORMS 4 and 5<br>**1. Positive nasopharyngeal swab**<br>**2. Very severe/severe clinical presentation**<br>**3. Onset or development of ARDS**<br>**4. Cytokine storm** (deterioration of general health status with ↑CRP, ↑fibrinogen, ↑D-dimer, ↑IL-6)	**Covid centers**: intensive care units	**All available** measures/combinations of measures need to be applied<br>**Maintaining ↑ O_2 flow** as long as possible, **MV** in case of further deterioration<br>**Corticosteroid therapy:** methylprednisolone 1-2 mg/kg, 3-5 days, then gradual reducing of the dose (dose should be adjusted to body mass in order to reduce the risk of bleeding and other adverse effects)<br>**Tocilizumab:** 8 mg/kg IV per dose. Two doses (max 800 mg per dose)<br>**Immunoglobulins:** 10 – 20 g per day Ig over a period of 3 – 5 days

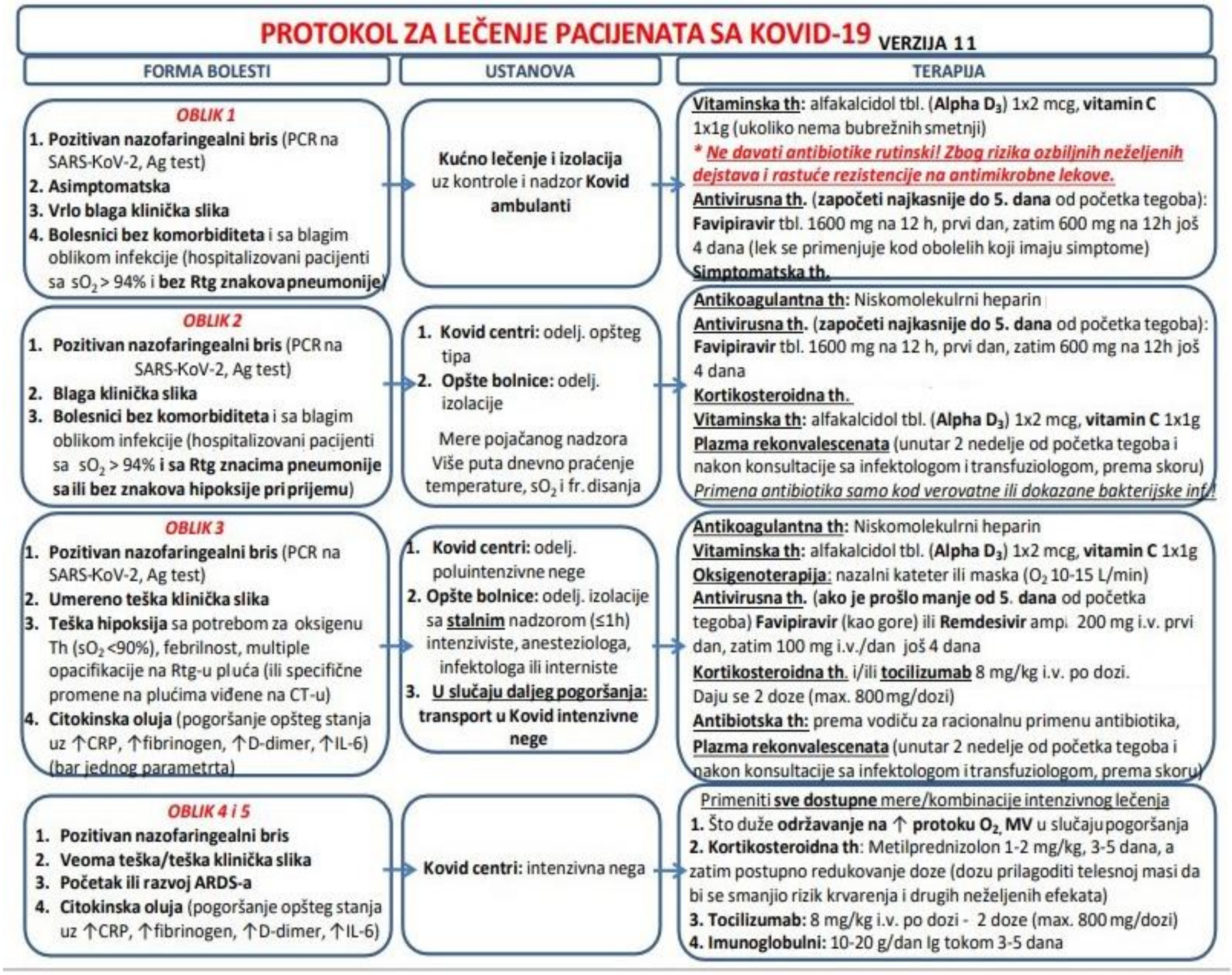
PROTOKOL ZA LEČENJE PACIJENATA SA KOVID-19 VERZIJA 11
FORMA BOLESTI
USTANOVA
TERAPIJA
OBLIK 1
1. Pozitivan nazofaringealni bris (PCR na SARS-KoV-2, Ag test)
2. Asimptomatska
3. Vrlo blaga klinička slika
4. Bolesnici bez komorbiditeta i sa blagim oblikom infekcije (hospitalizovani pacijenti sa sO2 > 94% i bez Rtg znakova pneumonije)
Kućno lečenje i izolacija uz kontrole i nadzor Kovid ambulanti
Vitaminska th: alfakalcidol tbl. (Alpha D3) 1x2 mcg, vitamin C 1x1g (ukoliko nema bubrežnih smetnji)
* Ne davati antibiotike rutinski! Zbog rizika ozbiljnih neželjenih dejstava i rastuće rezistencije na antimikrobne lekove.
Antivirusna th. (započeti najkasnije do 5. dana od početka tegoba): Favipiravir tbl. 1600 mg na 12 h, prvi dan, zatim 600 mg na 12h još 4 dana (lek se primenjuje kod obolelih koji imaju simptome)
Simptomatska th.
OBLIK 2
1. Pozitivan nazofaringealni bris (PCR na SARS-KoV-2, Ag test)
2. Blaga klinička slika
3. Bolesnici bez komorbiditeta i sa blagim oblikom infekcije (hospitalizovani pacijenti sa sO2 > 94% i sa Rtg znacima pneumonije sa ili bez znakova hipoksije pri prijemu)
1. Kovid centri: odelj. opšteg tipa
2. Opšte bolnice: odelj. izolacije
Mere pojačanog nadzora Više puta dnevno praćenje temperature, sO2 i fr. disanja
Antikoagulantna th: Niskomolekulrni heparin
Antivirusna th. (započeti najkasnije do 5. dana od početka tegoba): Favipiravir tbl. 1600 mg na 12 h, prvi dan, zatim 600 mg na 12h još 4 dana
Kortikosteroidna th.
Vitaminska th: alfakalcidol tbl. (Alpha D3) 1x2 mcg, vitamin C 1x1g
Plazma rekonvalescenata (unutar 2 nedelje od početka tegoba i nakon konsultacije sa infektologom i transfuziologom, prema skoru)
Primena antibiotika samo kod verovatne ili dokazane bakterijske inf.!
OBLIK 3
1. Pozitivan nazofaringealni bris (PCR na SARS-KoV-2, Ag test)
2. Umereno teška klinička slika
3. Teška hipoksija sa potrebom za oksigenu Th (sO2 <90%), febrilnost, multiple opacifikacije na Rtg-u pluća (ili specifične promene na plućima viđene na CT-u)
4. Citokinska oluja (pogoršanje opšteg stanja uz ↑CRP, ↑fibrinogen, ↑D-dimer, ↑IL-6) (bar jednog parametrta)
1. Kovid centri: odelj. poluintenzivne nege
2. Opšte bolnice: odelj. izolacije sa stalnim nadzorom (≤1h) intenziviste, anesteziologa, infektologa ili interniste
3. U slučaju daljeg pogoršanja: transport u Kovid intenzivne nege
Antikoagulantna th: Niskomolekulrni heparin
Vitaminska th: alfakalcidol tbl. (Alpha D3) 1x2 mcg, vitamin C 1x1g
Oksigenoterapija: nazalni kateter ili maska (O2 10-15 L/min)
Antivirusna th. (ako je prošlo manje od 5. dana od početka tegoba) Favipiravir (kao gore) ili Remdesivir ampl. 200 mg i.v. prvi dan, zatim 100 mg i.v./dan još 4 dana
Kortikosteroidna th. i/ili tocilizumab 8 mg/kg i.v. po dozi. Daju se 2 doze (max. 800 mg/dozi)
Antibiotska th: prema vodiču za racionalnu primenu antibiotika,
Plazma rekonvalescenata (unutar 2 nedelje od početka tegoba i nakon konsultacije sa infektologom i transfuziologom, prema skoru)
OBLIK 4 i 5
1. Pozitivan nazofaringealni bris
2. Veoma teška/teška klinička slika
3. Početak ili razvoj ARDS-a
4. Citokinska oluja (pogoršanje opšteg stanja uz ↑CRP, ↑fibrinogen, ↑D-dimer, ↑IL-6)
Kovid centri: intenzivna nega
Primeniti sve dostupne mere/kombinacije intenzivnog lečenja
1. Što duže održavanje na ↑ protoku O2, MV u slučaju pogoršanja
2. Kortikosteroidna th: Metilprednizolon 1-2 mg/kg, 3-5 dana, a zatim postupno redukovanje doze (dozu prilagoditi telesnoj masi da bi se smanjio rizik krvarenja i drugih neželjenih efekata)
3. Tocilizumab: 8 mg/kg i.v. po dozi - 2 doze (max. 800 mg/dozi)
4. Imunoglobulni: 10-20 g/dan Ig tokom 3-5 dana
